# A Novel Zein-Based Composite Nanoparticles for Improving Bioaccessibility and Anti-Inflammatory Activity of Resveratrol

**DOI:** 10.3390/foods10112773

**Published:** 2021-11-11

**Authors:** Jiaping Liu, Yaqiong Zhang, Wenwen Liu, Boyan Gao, Liangli (Lucy) Yu

**Affiliations:** 1 Institute of Food and Nutraceutical Science, School of Agriculture and Biology, Shanghai Jiao Tong University, Shanghai 200240, China; liujiaping@sjtu.edu.cn (J.L.); triplewen@sjtu.edu.cn (W.L.); gaoboyan@sjtu.edu.cn (B.G.); 2 China-Canada Joint Lab of Food Nutrition and Health (Beijing), Beijing Technology & Business University (BTBU), Beijing 100048, China; 3 Department of Nutrition and Food Science, University of Maryland, College Park, MD 20742, USA; lyu5@umd.edu

**Keywords:** zein, cross-linked sodium caseinate, resveratrol, nanoparticles, bioaccessibility, anti-inflammatory activity

## Abstract

A microbial transglutaminase-induced cross-linked sodium caseinate (MSC) was used to stabilize zein nanoparticles, and the study was to investigate whether zein-MSC nanoparticles (zein-MSC NPs) can be used as an encapsulation carrier for resveratrol. A group of resveratrol-loaded zein-MSC nanoparticles (Res-zein-MSC NPs) with varying zein to Res mass ratios was first prepared. The particle sizes and zeta-potentials were in the ranges from 215.00 to 225.00 nm and from −29.00 to −31.00 mV. The encapsulation efficiency (EE) of Res was also influenced by the zein to Res mass ratio, and the encapsulated Res existed in an amorphous form. The major interactions between Res and zein-MSC NPs were hydrogen bonding and hydrophobic interaction. Furthermore, compared with free Res, the photo-stability and bioaccessibility of Res-zein-MSC NPs were significantly improved. The cellular studies also showed that Res-zein-MSC NPs exhibited lower cytotoxicity and desirable anti-inflammatory activity.

## 1. Introduction

Resveratrol (Res) is a phenolic compound mainly found in berries, grapes, peanuts and pistachios [1]. It is widely noted for its biological activities, for example, the antioxidant [2] and anti-inflammatory activity [3]. However, Res has a poor water solubility and could undergo epimerization or degradation during food processing and storage. Its biological functions are often altered under physiological conditions, which largely limits its application in food industries as a nutraceutical or health ingredient [4]. Recently, some nano-sized carrier structures have been tailored towards the encapsulation and delivery of Res in its chemically active form to improve its physicochemical property and biological efficacy [5,6]. Among them, food protein-based nanoparticles seem to have more advantageous since food proteins are highly biocompatible and have special colloidal properties and molecular interactions with bioactive compounds [7,8].

Zein, a GRAS food-grade ingredient from corn, has a high hydrophobicity and desirable self-assembly property in water. It has been commonly used in the incorporation and delivery of hydrophobic bioactive components by forming nanoparticle aqueous dispersions [9]. Wang et al. used a low-energy anti-solvent method to encapsulate β-carotene into zein nanoparticles [10]. Nevertheless, zein nanoparticles has poor physical stability at neutral aqueous solutions since its isoelectric point was near 6.2 [11]. Therefore, it will influence the delivery behavior of the encapsulated bioactive compound. Several synthetic or natural molecules have been tested in different systems for their potential to stabilize zein nanoparticles [12,13]. The physico-chemical stability and re-dispersibility in water of zein-caseinate nanoparticles were improved in our previous study, due to the electrostatic and steric stabilization of sodium caseinate molecules adsorbed around zein nanoparticles [14]. However, another study showed that zein-caseinate nanoparticles were still susceptible during storage. It was found that their particle sizes significantly increased after 5 days of storage at 25 °C [15]. In order to achieve better stability effect, microbial transglutaminase-induced cross-linked sodium caseinate (MSC) was recently synthesized and tested for its potential as a novel stabilizer of zein nanoparticles by our group. Zein-MSC nanoparticles showed better colloidal stability characteristics, especially the storage stability compared to that of zein-caseinate nanoparticles. It was observed that the size and size distribution of zein-MSC nanoparticles did not change significantly, even over the 6 weeks of storage, which was attributed to the more stable network structure of MSC adsorbed around zein nanoparticles [16]. 

As a continuation, this study investigated whether zein-MSC nanoparticles can potentially be used as a carrier for encapsulation and releasing Res. First, Res-loaded zein-MSC nanoparticles were fabricated, and their physicochemical properties were characterized. The physical state of Res and its intermolecular interactions with carrier proteins were investigated by XRD, ATR-FTIR and fluorescence spectroscopy. The in vitro release behavior of Res-loaded zein-MSC nanoparticles was also examined. Most importantly, the photo-stability, bioaccessibility, and anti-inflammatory activity of Res were investigated before and after its encapsulation.

## 2. Materials and Methods

### 2.1. Chemicals

Trans-resveratrol (Res, 98% purity) and sodium caseinate (87% purity) were obtained from Sigma-Aldrich Chemical Co., Ltd. (St. Louis, MO, USA). Microbial transglutaminase (100 U/g) was supported by Jiangsu Yiming Biological Co., Ltd. (Taizhou, Jiangsu, China). Zein (97% protein content) was bought from Showa Sangyo (Tokyo, Japan).

### 2.2. Preparation of Res-Zein-MSC NPs

MSC and Res-zein-MSC NPs were prepared following previously reported laboratory procedures, respectively [14,16]. Briefly, MSC was synthesized under the optimized conditions: 50 mg/mL of sodium caseinate, 250 U/g of MTGase and 2 h of reaction time. Then, four zein/Res stock solutions (zein to Res mass ratios of 10:1, 8:1, 6:1, 4:1) were obtained by dissolving zein and Res in 80% (*v*/*v*) ethanol aqueous solutions. 20 mg/mL of zein was constant in four stock solutions. Then, the stock solution was dropped into MSC aqueous solution (8 mg/mL) under magnetic stirring, respectively. Zein-MSC nanoparticles without Res (zein-MSC NPs) were prepared under the same procedure as the blank. All the procedures using Res were carried out in dark in order to avoid its isomerization.

### 2.3. Particle Size, Size Distribution and Zeta-Potential

The particle size, size distribution (polydispersity index, PDI) and zeta-potential of zein-MSC NPs and Res-zein-MSC NPs were analyzed by dynamic light scattering technique at 25 °C.

### 2.4. Encapsulation Efficiency (EE)

The EE of Res-zein-MSC NPs was measured following a laboratory procedure [17]. After lyophilization, ethyl acetate was added to the freeze-dried Res-zein-MSC NPs, vortexed and centrifuged to wash away the unencapsulated Res. The supernatants were gathered after three washing processes. After that, a nitrogen evaporator was used to remove ethyl acetate from the supernatant. Methanol (1 mL) was used to re-dissolve unencapsulated Res, and an ultra-performance liquid chromatography (UPLC) with a PDA detector was used to measure the amount of Res. The mobile phase was ultra-pure water containing trifluoroacetic acid (0.1%, *v*/*v*) and acetonitrile with a volume ratio of 70:30. The detection wavelength was 306 nm. The Res concentration was calculated by calibration curve (R^2^ = 0.999). The EE of Res was obtained according to the equation shown below:

EE (%) = (1 − amount of free Res/total amount of Res) × 100(1)

### 2.5. Re-Dispersibility

The freeze-dried Res-zein-MSC NPs were re-dispersed in ultra-pure water to the original concentration under magnetic stirring. Then, particle samples without visible precipitations were characterized for the size, size distribution and zeta-potential.

### 2.6. Morphological Observations

Scanning electron microscopy (SEM) and transmission electron microscopy (TEM) were used to observe the microstructures of Res-zein-MSC NPs.

### 2.7. ATR-FTIR

Res, physical mixtures of Res and zein-MSC NPs, and Res-zein-MSC NPs with different zein to Res mass ratios were analyzed using an ATR-FTIR spectrophotometer. The scanning range was 600–4000 cm^−1^.

### 2.8. Fluorescence Spectroscopy

Intrinsic fluorescence of zein-MSC NPs and Res-zein-MSC NPs was recorded at 298, 310 and 315 K by using a multilabel plate reader. The excitation wavelength was set at 280 nm and the emission wavelength was set from 305 to 500 nm [18]. Besides, double-logarithmic and Van’t Hoff equations were established to calculate the thermodynamic parameters:

log (*F*_0_ − *F*/*F*) = log *K_a_* + *n* log [*Q*] (2)

ln *K_a_* = −∆*H/RT* + ∆*S/R* (3)

Δ*G* = Δ*H* − *T*Δ*S* (4)

*F*_0_ and *F* means the fluorescence intensities before and after Res encapsulation. [*Q*] means the Res concentration. *K_a_* is the binding constant and *n* is the number of binding sites. *R* means the gas constant and *T* is temperature. Δ*H*, Δ*S* and Δ*G* means enthalpy, entropy and Gibbs free energy change, respectively.

### 2.9. XRD

A poly-functional X-ray diffractometer with 2θ angle of 10–50° was used to capture the XRD patterns of Res, physical mixtures of Res and zein-MSC NPs, and Res-zein-MSC NPs.

### 2.10. Photo-Stability

Ultraviolet radiation at 365 nm was used to irradiate free Res (dissolved in 80% aqueous ethanol solution) and Res-zein-MSC NPs. The UPLC procedure described in Section 2.4. was used to measure the remained amount of Res at the time of 1, 2, 4, 8 and 12 h. The result is represented by the percentage of the remained Res.

### 2.11. Release Behavior

The release behaviors of free Res (dissolved in 80% aqueous ethanol solution) and freshly prepared Res-zein-MSC NPs aqueous dispersions was examined by the dialysis method [19]. The release behavior of Res was first determined in simulated gastric fluid (SGF, pH = 1.2) for 2 h, followed by 2 h in simulated intestinal fluid (SIF, pH = 7.4). To ensure the sink condition, tween 80 (1%, *w*/*v*) was added in the release media. Briefly, the dialysis bag containing 3 mL of free Res solution or Res-zein-MSC NPs aqueous dispersion was immersed in fresh release media and incubated in a shaker at 37 °C. At the time of 0.5, 1, 1.5, 2, 3 and 4 h, 1 mL of the release medium was collected and an equal volume of fresh medium was added each time. The UPLC method as described in Section 2.4. was used to measure the concentration of Res released into the medium. 

The release mechanism of Res was analyzed by the Korsmeyer-Peppas equation shown below [20]:

*M_t_/M_∞_* = *K*_KP_ × *t^n^* (5)

*M_t_/M_∞_* means the Res release fraction at time *t*, *K*_KP_ is a constant, and *n* means the release exponent [21].

### 2.12. Bioaccessibility

The bioaccessibility of free Res and freshly prepared Res-zein-MSC NPs aqueous dispersions were examined according to a previously reported protocol [22]. Briefly, 5 mL of free Res solution (dissolved in 80% aqueous ethanol solution) or Res-zein-MSC NPs aqueous dispersion was mixed with 5 mL of SGF (pH = 1.2) and incubated in a shaker at 37 °C for 2 h. Then, 10 mL of SIF (pH = 7.4) was added and incubated together for another 2 h. Finally, the raw digestive juice was centrifuged at 10,000 rpm for 1 h, and the micelle phase (clear supernatant) was collected. The bioaccessibility (%) of Res was calculated using the equation shown below:

bioaccessibility (%) = Res in micelle phase/Res before digestion × 100(6)

### 2.13. Cytotoxicity Assay

#### 2.13.1. Cell Culture

The RAW 264.7 cells were cultured in DMEM medium at 37 °C in a CO_2_ incubator.

#### 2.13.2. MTT Assay

The effects of free Res and Res-zein-MSC NPs on the viability of RAW 264.7 cells were assessed using an MTT metabolic activity assay. Briefly, RAW 264.7 cells (20,000 cells/well) were treated with free Res or Res-zein-MSC NPs (5, 10, 20, 30, 40 and 50 μg/mL) in the fresh DMEM medium with 0.1% (*v*/*v*) DMSO for 24 h. Then, 100 μL of MTT solution was added and incubated for another 4 h in dark. Centrifugation at 3000 rpm for 5 min was used to remove the media and 150 μL of DMSO was added to obtain the absorbance at 490 nm. The cell viability was calculated shown below:

cell viability (%) = A_t_/A_b_ × 100 (7)

Here, A_t_ and A_b_ are the absorbance of the treated and blank cell dispersion, respectively.

### 2.14. Anti-Inflammatory Activity

RAW 264.7 cells (20,000 cells/well) were treated with Res-zein-MSC NPs (5, 10, 20, 30, 40 and 50 μg/mL) for 1 h. 1 μL of LPS (0.1 mg/mL) was added and incubated for another 24 h. The supernatants were obtained after centrifugation, and the inflammatory mediators of NO, IL-1β and IL-6 were measured using their test kits. Briefly, the Griess Reagent method was used to detect the level of NO and the absorbance was determined at 540 nm. The ELISA method was used to detect levels of IL-1β and IL-6, and the absorbance was both determined at 450 nm.

### 2.15. Statistical Analysis

Mean ± SD was used to present the data for triplicate measurements. One-way ANOVA and *Tukey’s* test by SPSS were used to identify the mean differences. *p* < 0.05 indicates a statistical significance.

## 3. Results and Discussion

### 3.1. Characterization of Res-Zein-MSC NPs

Res-zein-MSC NPs with zein to Res mass ratios of 10:1, 8:1, 6:1 and 4:1 were fabricated and their characteristic data are shown in Table 1. All the freshly prepared Res-zein-MSC NPs aqueous dispersions had a pH range of 6.53~6.64. The particle sizes and zeta-potentials were in the range of 216.43 to 221.00 nm and −29.93 to −30.70 mV, respectively, without significant differences (*p* > 0.05). The polydispersity index (PDI) of Res-zein-MSC NPs were all around 0.2, indicating a monodispersed distribution. These results indicated that the zein to Res mass ratio had no influence on the size, size distribution and zeta-potential of nanoparticles. Furthermore, the EE is a fundamental index to evaluate the loading capability of a delivery system [23]. The EE of Res-zein-MSC NPs with zein to Res mass ratios of 10:1, 8:1 and 6:1 was 81.93, 80.14 and 73.54%, respectively, with no significant differences (*p* > 0.05) (Table 1). However, when the zein to Res mass ratio further decreased to 4:1, the EE of nanoparticles significantly decreased to 58.90% (*p* < 0.05). Compared with some previous nano-delivery systems for resveratrol, the encapsulation efficiencies of Res-zein-MSC NPs were comparable or greater, suggesting that zein-MSC NPs was an efficient delivery carrier for Res [24,25].

**Figure 1:**
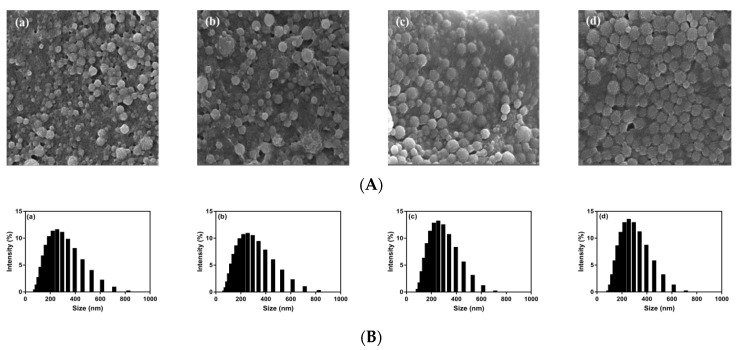
SEM images (**A**) and particle size distributions (**B**) of Res-zein-MSC NPs at zein to Res mass ratios of 10:1 (**a**), 8:1 (**b**), 6:1 (**c**) and 4:1 (**d**), respectively.

The morphology of Res-zein-MSC NPs was observed by SEM (Figure 1A). All the Res-zein-MSC NPs showed the spherical shape, with their diameters ranging from 125.00 to 275.00 nm. These observations were consistent with the results determined by the DLS technique (Figure 1B and Table 1). Moreover, the surfaces of Res-zein-MSC NPs with zein to Res mass ratios of 10:1 and 8:1 seemed smooth, whereas a relatively rougher surface was observed for the nanoparticles with zein to Res mass ratios of 6:1 and 4:1. This phenomenon may be due to the relatively lower EE of Res-zein-MSC NPs with zein to Res mass ratios of 6:1 and 4:1 (Table 1), and there was a certain amount of unencapsulated Res on the surfaces of nanoparticles [26].

When all the freeze-dried Res-zein-MSC NPs were re-dispersed in water, no visible aggregates were observed, and uniform dispersions were formed. In comparison, free Res with the same amount could not be dissolved in water due to its low water solubility [27], and visible sediments were shown at the bottom of vials (Figure S1). Besides, the sizes of the re-dispersed Res-zein-MSC NPs ranged from 167.30 to 172.63 nm with a uniform size distribution (PDI < 0.25) (Table 1). A similar phenomenon was also observed in a previous literature whereby the particle size of curcumin-loaded gliadin-rhamnolipid composite nanoparticles decreased after re-dispersion [28]. The absolute zeta-potential values of Res-zein-MSC NPs increased after re-dispersion (−35.27~−38.87 mV), showing a stronger electrostatic repulsion among the re-dispersed nanoparticles [29]. The changes in particle size and zeta-potential after re-dispersion might suggest the occurrence of intermolecular rearrangement in Res-zein-MSC NPs.

### 3.2. ATR-FTIR

The FTIR spectra and characteristic peak assignments of Res, physical mixtures of Res and zein-MSC NPs, and Res-zein-MSC NPs are shown in Figure S2 and Table S1, respectively. The band intensity at 3000–3500 cm^−1^ contributing to the stretching vibration of OH group in Res was decreased in the spectra of Res-zein-MSC NPs compared to those of free Res. This suggested a possible intermolecular hydrogen bonding interaction between Res and carrier proteins [30]. Meanwhile, Res and physical mixture of Res and zein-MSC NPs both showed several characteristic peaks of Res in the range of 1700–600 cm^−1^, which had disappeared in the spectra of Res-zein-MSC NPs (Table S1). For example, the characteristic peaks of Res at 1008, 986 and 963 cm^−1^ reflecting trans olefinic H-C=C-H bending vibrations had disappeared after encapsulation, also indicating the existence of hydrophobic interactions between Res and carrier proteins.

### 3.3. Fluorescence Spectroscopy

The fluorescence spectra of zein-MSC NPs at 298, 310 and 315 K showed that zein-MSC NPs had a maximum fluorescence emission peak at 315 nm (Figure S3), which may be attributed to the high content of tyrosine residues in zein (about 5.0% *w*/*w*) [12]. However, the characteristic emission peak of zein was almost quenched and a new emission peak appeared near 383 nm for Res-zein-MSC NPs, which may be related to the characteristic emission peak of Res [18]. Moreover, the fluorescence intensity of Res-zein-MSC NPs decreased and its maximum emission peak also shifted from 383 nm to 391 nm with decreasing zein to Res mass ratio from 10:1 to 4:1. The observed red shift of the maximum emission peak of Res-zein-MSC NPs suggested the increased polar micro-environment of proteins, possibly due to the increased hydrophobic interactions between Res and carrier proteins [31].

Several thermodynamic parameters calculated from the fluorescence spectra can also indicate the intermolecular interactions between Res and carrier proteins, including ΔH, ΔS and ΔG. It was shown that the values of ΔH and ΔS were −14.9743 kJ/mol and −6.2854 J/mol/K, and the values of ΔG were also negative at 298, 310 and 315 K, respectively (Table S2), suggesting a spontaneous binding of Res with the carrier proteins [32]. According to the theory of Ross and Olsson, hydrogen bonding was dominant in the binding reaction (ΔH < 0, ΔS < 0) [33]. Taking the fluorescence spectroscopy results together, hydrogen bonding and hydrophobic interaction were found to be the major intermolecular interactions between Res and the carrier proteins, which was consistent with the FTIR observations in this study (Figure S2).

### 3.4. XRD

In the XRD pattern of Res, multiple characteristic peaks (13.26°, 16.36°, 19.20°, 20.30°, 22.30°, 23.48°, 25.26° and 28.28°) were observed (Figure S4), indicating the crystalline state of free Res [34]. Meanwhile, these peaks also appeared in the XRD pattern of physical mixtures of Res and zein-MSC NPs. In contrast, all these characteristic peaks of Res could not be observed for Res-zein-MSC NPs, indicating the amorphous status of Res after encapsulation [35].

### 3.5. Photo-Stability Study

**Figure 2:**
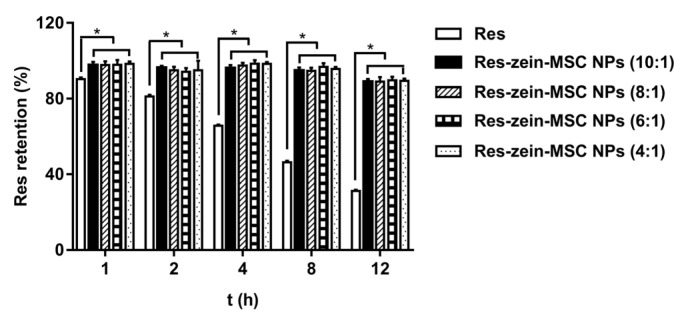
The photo-stability of Res and Res-zein-MSC NPs under UV radiation. Here, * represents a significant difference between samples (*p* < 0.05).

Res is known to be highly sensitive to ultraviolet (UV) radiation, and it could undergo chemical epimerization and degradation under UV light [36]. Therefore, the photo-stability of Res was investigated and compared before and after the encapsulation. Due to its poor photo-stability, only 90.27, 81.15, 65.69, 46.37 and 31.29% of free Res was retained after 1, 2, 4, 8 and 12 h of UV exposure, respectively (Figure 2). However, the encapsulated Res in nanoparticles remained stable after 12 h of UV radiation and 88.86–89.64% of Res was retained in zein-MSC NPs with different mass ratios of zein to Res. These results suggested that zein-MSC NPs was able to protect Res against UV radiation, agreeing to several previous results that the photo-stability of Res was improved after encapsulation into protein-based nanoparticles [24,37]. These observations might be explained by the fact that the UV light was partly absorbed by aromatic groups and double bonds in proteins, and meanwhile the protein-based nanoparticles could also scatter UV light more efficiently [4]. Meanwhile, the small loss of Res-zein-MSC NPs observed under UV radiation may be due to the degradation of unencapsulated Res on the surfaces of nanoparticles.

### 3.6. Release Behavior

**Figure 3:**
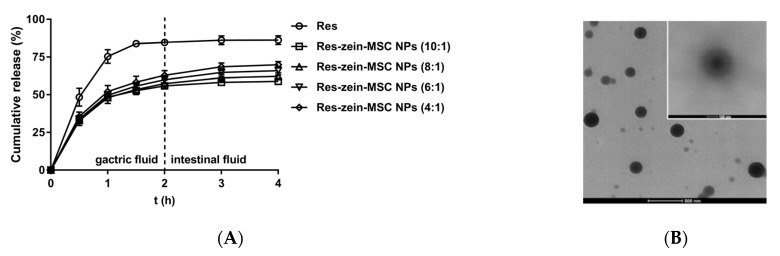
The release profiles of Res and Res-zein-MSC NPs (**A**); TEM images of Res-zein-MSC NPs before and after (upper right corner) pepsin digestion (**B**).

The release kinetics of free Res and encapsulated Res from zein-MSC NPs were compared and are shown in Figure 3A. A burst release was found for free Res with 48.35% release after 0.5 h and 75.35% release after 1 h in SGF. In contrast, a slower and sustained release behavior was observed for Res-zein-MSC NPs. The cumulative release for four kinds of Res-zein-MSC NPs were in the range of 32.85–35.66% and 48.06–52.13% after 0.5 h and 1 h in SGF, respectively. These results indicated the efficient encapsulation of Res inside the nanoparticles [38]. Besides, significant differences in cumulative release were observed among free Res and four kinds of Res-zein-MSC NPs both in SGF and SIF (*p* < 0.05). For the four kinds of Res-zein-MSC NPs, the cumulative release of Res-zein-MSC NPs with zein to Res mass ratio of 4:1 was significantly higher than those of other three Res-zein-MSC NPs in SIF (*p* < 0.05). Meanwhile, the release of Res from zein-MSC NPs was much faster in SGF than that in SIF. This phenomenon might be due to that the carrier protein zein and MSC could be partly digested by pepsin in SGF, thus inducing a faster release of the encapsulated Res. The explanation was also verified by the TEM results (Figure 3B). Compared with the TEM image of Res-zein-MSC NPs before release, the looser structure and unclear boundary of nanoparticles were observed after a 2 h release (inserted image in the upper right corner), possibly due to the digestion of carrier proteins in SGF. Besides, the release exponent n was analyzed using the Korsmeyer-Peppas model (Table S3). The n values of Res-zein-MSC NPs were all below 0.43, suggesting a pseudo-Fickian diffusion-controlled release mechanism of Res from zein-MSC NPs [39]. In brief, zein-MSC NPs may achieve a sustained Res delivery in stomach, but further modifications of the nanoparticles are needed to afford an intestinal delivery of Res or other non-polar bioactive components.

### 3.7. Bioaccessibility

**Figure 4:**
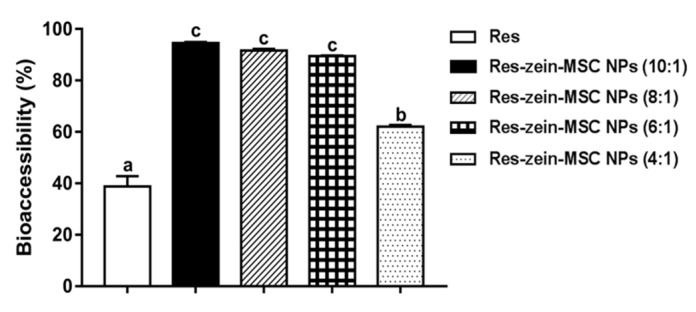
The bioaccessibility of Res and Res-zein-MSC NPs. Here, different letters represent significant differences (*p* < 0.05).

The bioaccessibility of free Res was only 38.76%. In comparison, the bioaccessibilities of Res-zein-MSC NPs with zein to Res mass ratios of 10:1, 8:1, 6:1 and 4:1 significantly increased to 94.57, 91.65, 89.44 and 62.03%, respectively (*p* < 0.05) (Figure 4), which might be attributed to the increased aqueous solubility [24] and amorphous state of Res in zein-MSC NPs (Figure S4) [34,40]. Moreover, there were no significant differences in the bioaccessibilities of Res-zein-MSC NPs with zein to Res mass ratios of 10:1, 8:1 and 6:1, but the bioaccessibility of Res-zein-MSC NPs with zein to Res mass ratio of 4:1 was significantly lower (*p* < 0.05). This result might be due to the significantly lower encapsulation efficiency of nanoparticles with zein to Res mass ratio of 4:1, and a greater amount of unencapsulated Res might adsorb on the surface of the nanoparticles (Table 1 and Figure 1A).

### 3.8. Cytotoxicity Assay

**Figure 5:**
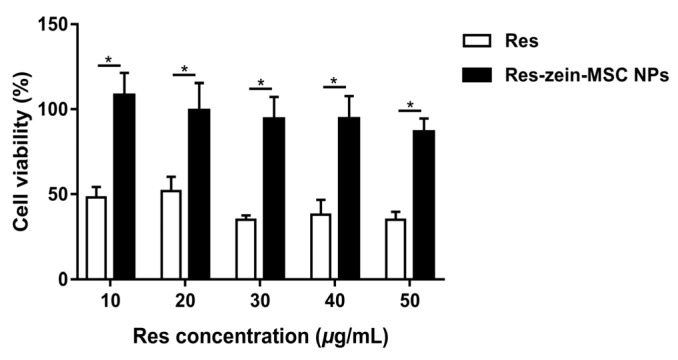
The cell viabilities of RAW 264.7 cells after treating with Res and Res-zein-MSC NPs. Here, * represents a significant difference between samples (*p* < 0.05).

The cellular toxicity of free Res and Res-zein-MSC NPs were tested at different concentrations of Res by MTT assay in the cultured RAW 264.7. The cell viability treated with free Res at the concentration of 10, 20, 30, 40 and 50 μg/mL was 48.08, 51.75, 35.10, 38.09 and 35.09%, respectively, showing the obvious cellular toxicity (Figure 5). In contrast, Res-zein-MSC NPs with the same Res concentrations all resulted in an above 80% cell viability under the same experimental conditions, indicating the negligible cytotoxicity of Res-zein-MSP NPs. These observations suggested that the encapsulation of Res into zein-MSC NPs can significantly reduce its cytotoxicity, which might be due to the highly compatibility of protein carriers in Res-loaded nanoparticles [41].

### 3.9. Anti-Inflammatory Activity

**Figure 6:**
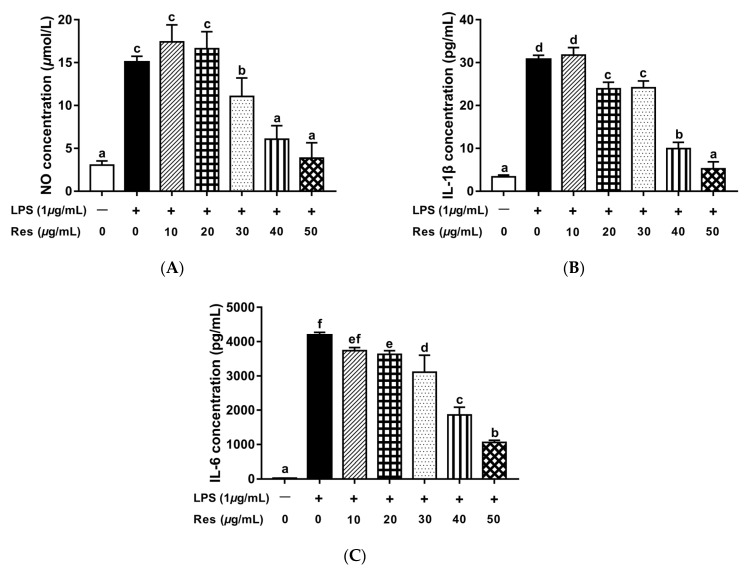
The effect of Res-zein-MSC NPs treatment on the secretion levels of NO (**A**), IL-1β (**B**) and IL-6 (**C**) in LPS-induced RAW 264.7 cells. Here, different letters represent significant differences (*p* < 0.05).

The intracellular nitric oxide (NO) release and several critical proinflammatory cytokines of IL-1β and IL-6 are involved in the signal transduction of inflammatory responses, and the inhibition of their secretions may lead to alleviation of the inflammatory effects [5]. Since Res-zein-MSC NPs with Res concentrations of 10–50 μg/mL all showed negligible cytotoxicity against RAW 264.7 macrophage cells, their anti-inflammatory activities were further examined. Compared to the control group, the secreted levels of NO, IL-1β and IL-6 statistically increased after LPS stimulation (*p* < 0.05). Res-zein-MSC NPs dose-dependently reduced the levels of NO, IL-1β and IL-6 (Figure 6). Moreover, Res-zein-MSC NPs significantly decreased the secretion levels of IL-1β and IL-6 by about 22.13% and 13.28% at the Res concentration of 20 μg/mL, while a significant inhibition of NO release was observed at the Res concentration of 30 μg/mL (*p* < 0.05). Further increasing the concentration of Res to 50 μg/mL resulted in the decreased levels of NO, IL-1β and IL-6 to 3.85 μmol/L, and 5.22 and 1061.39 pg/mL, respectively. Taken together, these observations suggested that the encapsulation of Res in zein-MSC NPs could reduce its cytotoxicity, while retaining its anti-inflammatory activity.

## 4. Conclusions

Resveratrol-loaded zein-MSC nanoparticles (Res-zein-MSC NPs) with desirable physico-chemical characteristics were successfully prepared and evaluated. Compared to free Res, Res-loaded zein-MSC NPs had an improved water dispersibility, UV irradiation stability and bioaccessibility. In addition, the encapsulation was able to reduce the cytotoxicity of Res, while not losing its desirable anti-inflammatory activity. This study suggested a potential utilization of zein-MSC nanoparticles as a novel delivery carrier in food, supplemental or pharmaceutical products for encapsulation and delivery of hydrophobic bioactive compound.

## Figures and Tables

**Table 1:** Characterization of resveratrol-loaded zein-MSC nanoparticles *.

Zein:Res (*w*/*w*)	State of the Dispersion	pH of the Dispersion	Z-Average (nm)		PDI		Zeta-Potential (mV)		Encapsulation Efficiency (%)
			Before Lyophilization	Re-Dispersion	Before Lyophilization	Re-Dispersion	Before Lyophilization	Re-Dispersion	
10:1	uniform	6.53 ^a^ ± 0.02	216.43 ^a^ ± 2.06	172.63 ^b^ ± 1.15	0.20 ^a^ ± 0.01	0.21 ^a^ ± 0.01	−30.03 ^a^ ± 1.50	−35.27 ^b^ ± 0.47	81.93 ^b^ ± 2.01
8:1	uniform	6.55 ^a^ ± 0.02	220.03 ^ab^ ± 2.89	171.23 ^b^ ± 4.69	0.18 ^a^ ± 0.01	0.23 ^a^ ± 0.03	−30.70 ^a^ ± 0.26	−37.87 ^a^ ± 0.42	80.14 ^b^ ± 3.69
6:1	uniform	6.64 ^b^ ± 0.01	219.90 ^ab^ ± 1.32	168.67 ^ab^ ± 1.66	0.19 ^a^ ± 0.01	0.23 ^a^ ± 0.02	−29.93 ^a^ ± 0.57	−38.87 ^a^ ± 0.55	73.54 ^b^ ± 3.77
4:1	uniform	6.57 ^a^ ± 0.02	221.00 ^ab^ ± 3.06	167.30 ^ab^ ± 1.06	0.20 ^a^ ± 0.02	0.24 ^a^ ± 0.02	−30.33 ^a^ ± 0.67	−35.83 ^b^ ± 0.42	58.90 ^a^ ± 1.99

* Different superscript lowercase letters in the same column indicate significant differences (*p* < 0.05).

## Data Availability

Not applicable.

## Supplementary Materials

The following are available online at https://www.mdpi.com/article/10.3390/foods10112773/s1, Table S1: Peak assignments of Res, physical mixtures of Res and zein-MSC NPs and Res-zein-MSC NPs in FTIR spectra; Table S2: The thermodynamic parameters obtained from double-logarithmic and Van’t Hoff equations; Table S3: Parameters of the fitting curve of Korsmeyer-Peppas model in the study of in-vitro release behavior; Figure S1: Photo images of free Res and freeze-dried Res-zein-MSC NPs (zein to Res mass ratio of 10:1 (A), 8:1 (B), 6:1 (C) and 4:1 (D)), which are dispersed in water at the same amount of Res; Figure S2: FTIR spectra for Res (a), physical mixtures of Res and zein-MSC NPs (b), and Res-zein-MSC NPs at zein to Res mass ratios of 10:1 (c), 8:1 (d), 6:1 (e) and 4:1 (f), respectively; Figure S3: Emission fluorescence spectra for zein-MSC NPs and Res-zein-MSC NPs at 298 K (A), 310 K (B) and 315 K (C), Figure S4: XRD spectra for Res (a), physical mixtures of Res and zein-MSC NPs (b), and Res-zein-MSC NPs at zein to Res mass ratios of 10:1 (c), 8:1 (d), 6:1 (e) and 4:1 (f), respectively.
