# Supplementary material for: A Novel Zein-Based Composite Nanoparticles for Improving Bioaccessibility and Anti-Inflammatory Activity of Resveratrol
Source: Foods. 2021 Nov 11;10(11):2773. doi: 10.3390/foods10112773 (PMC8624517; doi:10.3390/foods10112773)
Supplement: Supplementary file 1 [file foods-10-02773-s001.zip › foods-1439996-supplementary.pdf]

Supplementary Materials

A Novel Zein-Based Composite Nanoparticles for Improving Bioaccessibility and Anti-Inflammatory Activity of Resveratrol

Liu Jiaping [1], Zhang Yaqiong [1,2,*], Liu Wenwen [1], Gao Boyan [1] and Yu Liangli (Lucy) [3]

[1] Institute of Food and Nutraceutical Science, School of Agriculture and Biology, Shanghai Jiao Tong University, Shanghai 200240, China;
[2] China-Canada Joint Lab of Food Nutrition and Health (Beijing), Beijing Technology & Business University (BTBU), Beijing 100048, China;
[3] Department of Nutrition and Food Science, University of Maryland, College Park, MD 20742, United States;
* Correspondence: yqzhang2006@sjtu.edu.cn; Tel.: 021-34204538

Table S1. Peak assignments of Res, physical mixtures of Res and zein-MSC NPs and Res-zein-MSC NPs in FTIR spectra.

Res (cm^{-1})	Physical mixtures of Res and zein-MSC NPs (cm^{-1})	Res-zein-MSC NPs (cm^{-1})	Peak assignments*
1604.56	1605.16	-	ν(aromatic -C-C-)
1582.75	1583.11	-	ν(olefinic -C=C-)
1509.41	1509.30	-	ν(aromatic ring -C=C-)
1461.52	1461.49	-	ν(benzene ring)
1440.28	1440.83	1447.89	ν(aromatic ring -C=C-)
1379.63	1380.11	-	ν(-C-O–)
1322.62	1322.44	-	δ(=C-H)
1143.93	1144.74	-	ν(Phenolic -C-O–)
1008.82	1008.98	-	δ(trans olefinic H-C=C-H)
986.17	986.51	-	δ(trans olefinic H-C=C-H)
963.71	964.18	-	ν(trans olefinic -C=C-)
828.09	828.86	-	δ(=C-H of phenolic ring)
673.30	673.70	-	δ(=C-H of trans double bond)
603.11	603.61	-	δ(=C-H of trans double bond)

* Vibration modes: ν, stretching; δ, bending.

Cited from:

1. Davidov-Pardo, G.; Joye, I.J.; McClements, D.J. Encapsulation of resveratrol in biopolymer particles produced using liquid antisolvent precipitation. Part 1: Preparation and characterization. *Food Hydrocoll*. **2015**, 45, 309-316.
2. Jangid, A.K.; Patel, K.; Jain, P.; Patel, S.; Gupta, N.; Pooja, D.; Kulharia, H. Inulin-pluronic-stearic acid based double folded nanomicelles for pH-responsive delivery of resveratrol. *Carbohydr. Polym*. **2020**, 247, 116730.
3. Khan, M.A.; Yue, C.; Fang, Z.; Hu, S.; Cheng, H.; Bakry, A.M.; Liang, L. Alginate/chitosan-coated zein nanoparticles for the delivery of resveratrol. *J. Food Eng*. **2019**, 258, 45-53.
4. Porto, I.C.C.M.; Nascimento, T.G.; Oliveira, J.M.S.; Freitas, P.H.; Haimeur, A.; França, R. Use of polyphenols as a strategy to prevent bond degradation in the dentin–resin interface. *Eur. J. Oral Sci.* **2018**, 126, 146-158.
5. Pushpalatha, R.; Selvamuthukumar, S.; Kilimozhi, D. Carbonyl and carboxylate crosslinked cyclodextrin as a nanocarrier for resveratrol: in silico, in vitro and in vivo evaluation. *J. Incl. Phenom. Macrocycl. Chem*. **2018**, 92, 261-272.
6. Qin, L.L.; Zhao, X.Y.; He, Y.W.; Wang, H.Q.; Wei, H.J.; Zhu, Q.; Zhang, T.; Qin, Y.; Du, A. Preparation, characterization, and in vitro evaluation of resveratrol-loaded cellulose aerogel. *Materials*. **2020**, 13, 1624.
7. Wei, Y.; Yu, Z.P.; Lin, K.S.; Sun, C.X.; Dai, L.; Yang, S.F.; Mao, L.k.; Yuan, F.; Gao, Y.X. Fabrication and characterization of resveratrol loaded zein-propylene glycol alginate-rhamnolipid composite nanoparticles: Physicochemical stability, formation mechanism and in vitro digestion. *Food Hydrocoll*. **2019**, 95, 336-348.

Table S2. The thermodynamic parameters obtained from double-logarithmic and Van't Hoff equations.

**T (K)**	**K_a (10^2 L mol^{-1})**	**n**	**r^2**	**ΔH (KJ mol^{-1})**	**ΔS (J mol^{-1} K^{-1})**	**ΔG (KJ mol^{-1})**
298	1.9467	0.8234	0.9958			-13.1004
310	1.6391	0.7488	0.9929	-14.9743	-6.2854	-13.0249
315	1.3763	0.7075	0.9841			-12.9935

Table S3. Parameters of the fitting curve of Korsmeyer-Peppas model in the study of *in-vitro* release behavior.

**Zein:Res (w/w)**	**n**	**r^2**
10:1	0.2575	0.8684
8:1	0.3000	0.9031
6:1	0.2987	0.9079
4:1	0.3267	0.9288

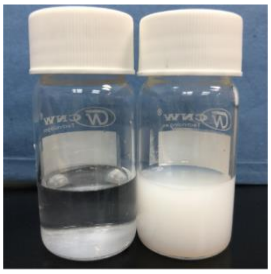

(A)

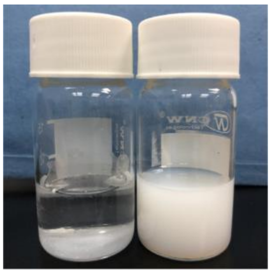

(B)

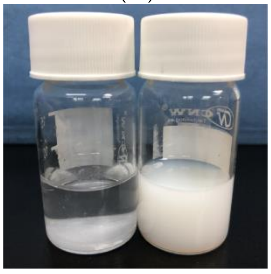

(C)

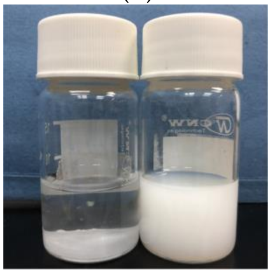

(D)

Figure S1. Photo images of free Res and freeze-dried Res-zein-MSC NPs (zein to Res mass ratio of 10:1 (A), 8:1 (B), 6:1 (C) and 4:1 (D)), which are dispersed in water at the same amount of Res.

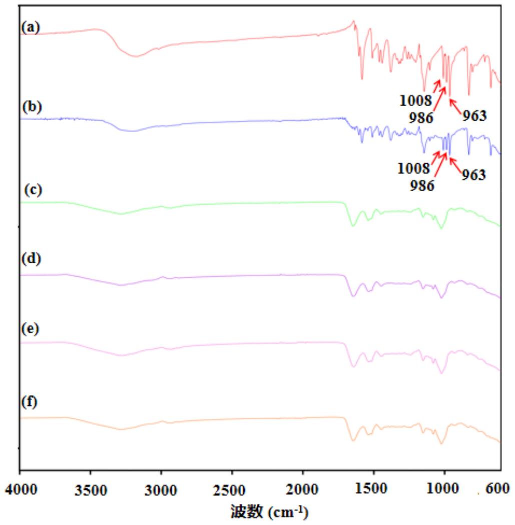

Figure S2. FTIR spectra for Res (a), physical mixtures of Res and zein-MSC NPs (b), and Res-zein-MSC NPs at zein to Res mass ratios of 10:1 (c), 8:1 (d), 6:1 (e) and 4:1 (f), respectively.

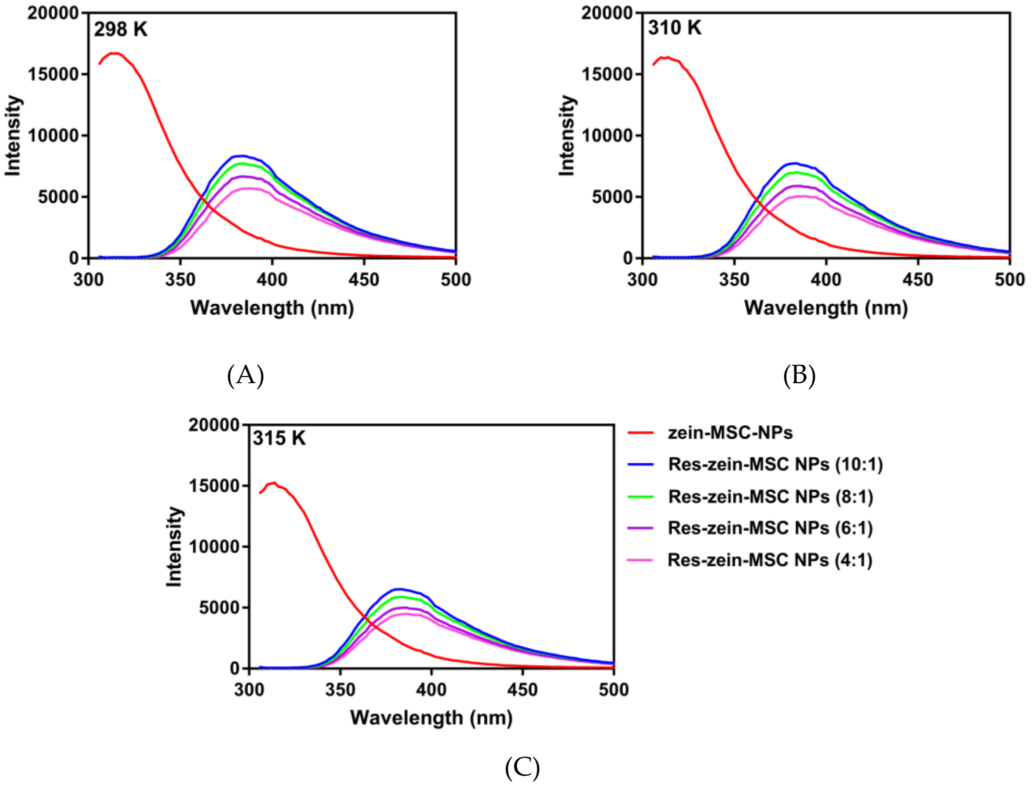

Figure S3. Emission fluorescence spectra for zein-MSC NPs and Res-zein-MSC NPs at 298 K (A), 310 K (B) and 315 K (C).

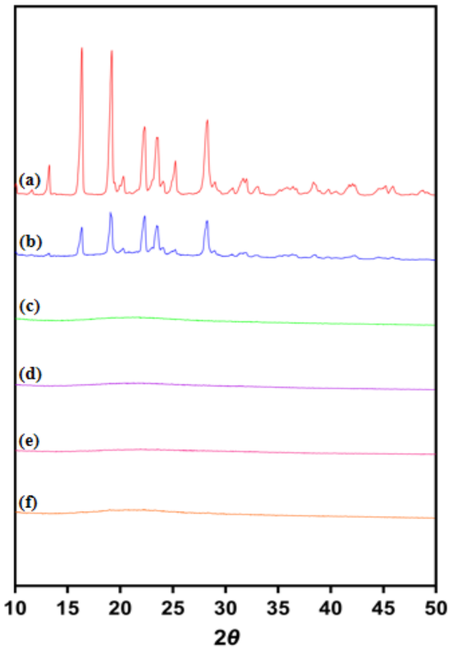

Figure S4. XRD spectra for Res (a), physical mixtures of Res and zein-MSC NPs (b), and Res-zein-MSC NPs at zein to Res mass ratios of 10:1 (c), 8:1 (d), 6:1 (e) and 4:1 (f), respectively.
